# Three Cases of Asthmatic Attacks, Accompanying Emphsema, Temporarily Relieved by Veratum Viride

**Published:** 1863-11

**Authors:** Geo. Winch

**Affiliations:** Otsego, Wisconsin


### THREE CASES OF ASTHMATIC ATTACKS,

### ACCOMPANYING EMPHSEMA, TEMPORARILY RELIEVED BY VERATUM VIRIDE.

By GEO. WINCH, M. D., of Otsego, Wisconsin.

In the summer of 1862, Miss Mary S., aged 16, good constitution, applied to me, stating she had attacks of great difficulty of breathing, lasting from two to four days; then expectoration commenced, discharge from the lungs freely, and would be relieved. On examination of the lungs in intervals of these attacks, I found expiration equal length of inspiration, no roles. I ordered her Sulph. Æth. and Tinct. Opii, and the latter separate in full doses, the smoking of Stramonium leaves, and different staple anodynes and anti-spasmodics. They relieved her but little. One day, as the attack was coming on, I gave her Veratrum Viride, Fluid Ext., gtt. iv. It relieved her in thirty minutes. Since that time, she keeps the Veratrum with her, and when she feels the difficulty approaching, she takes four drops, and now tells me she has not failed in checking the difficulty since I first used it.

Case 2nd.—Mr. A. D., aged 37, applied to me soon after I began the treatment of Case 1st. For a number of years he has suffered from repeated attacks of asthma, lasting two or three days and then, as in the former case, free expectoration and relief. In the intervals, inspiration was weak, followed by a prolonged blowing expiration. The breathing laborious, but slow. Heart sounds, normal. He told me that he had used all the anodynes and anti-spasmodics that different physicians had recommended him, but they did not prevent the attack, and only relieved him slightly. As he was attacked very severely one evening, I gave him Fluid Ext. Veratrum, gtt. v, which he said relieved him more than any medicine he had ever taken. In four hours he took four drops more, and the next day his breathing was as free as usual, and felt well. Now, when he feels the attack approaching, he takes five drops of the Veratrum, and the effect is as in the former case. Mr. D. has been a miner in California, and he tells me, eighteen months, while there, he was not troubled with the difficulty.

In the third case, the respiration was more normal in the intervals, but the attacks were frequent and severe, and relieved by Veratrum.

The pulse, in the first and second cases, beat ten or fifteen times less after using the medicine than before using it, at which time it was little above its normal standard.

I will say nothing of its action theoretically; but the clinical fact can be proven at any time by the patients.

